# Spine and TMJ: A Pathophysiology Report

**DOI:** 10.3390/jfmk5020024

**Published:** 2020-03-30

**Authors:** Luca Fiorillo

**Affiliations:** Department of Biomedical and Dental Sciences, Morphological and Functional Images, University of Messina, 98100 Messina, ME, Italy; lfiorillo@unime.it

**Keywords:** dentistry, TMJ, spine, anatomy, posturology, gnathology, orthodontics, physiology, pathology, jaws

## Abstract

The relationship between posture, spine, and temporomandibular joint (TMJ) is still a hotly debated topic in medicine. TMJ takes part in different physiological functions of the organism, starting from its embryological development, it is possible that it influences different vital functions. There is a strong connection between the respiratory tract and dental/maxillary occlusion or anatomy. The altered physiology of this district leads to pathologies that could affect the whole organism. On the contrary, it is also possible to highlight some symptoms of distant organism districts. Knowing well the pathophysiology of this district and semiotics, it is also possible to diagnose pathologies affecting other organs.

The relationship between malocclusion, body posture, and temporomandibular joint disorders (TMJ) is a controversial topic and still widely debated in the scientific field. A physiological occlusion is a condition in which there is a functional homeostatic balance between the various tissues/organs of the stomatognathic system. Teeth are subjected to biomechanical stresses that are normally dissipated without problems, since the occlusal and masticatory stress is counterbalanced by the ability to adapt the supporting tissues, muscles, and TMJ. The stomatognathic system, however, fits into other systems: swallowing, breathing, the cranio-sacral system, and the cranio-cervical posture, which could negatively influence the good functioning of chewing and cause a non-physiological occlusion. It is also the task of the osteopath to investigate these systems in the analysis of a manifest or latent malocclusion [1,2,3,4,5,6,7,8]. 

The tongue could be considered as a cephalic extension of the cervical spine. It originates from the four sub-occipital somites. With the development of the branchial arches of the maxilla and the jaw, these somites migrate anteriorly and give rise to the tongue. Embryology therefore explains the connections between the hypoglossal nerve and the cervical plexus and their functional synergy. During a physiological swallowing process, the fixation of the hyoid bone occurs without activation of the cervical musculature. From clinical observation, it could be observed that when swallowing is not correct, the involvement of this musculature is proportional to the degree of dysfunction, which should increase cervical lordosis. This phenomenon is a reflection of the extension of C0-C1-C2 (cervical vertebrae), the inclination of the Frankfurt plane (from back to front and from bottom to top), and the consequent loss of horizontality of the gaze. In order to recover the horizontal position, automatic compensations are implemented that pivot on C3-C4, with consequent reduction of cervical lordosis and excessive work of the cervical axial musculature. These are all possible predisposing factors for cervicalgia and muscle-tension headaches. Any dysfunction of the cervical plexus could retransmit on the phrenic nerve (C3-C4-C5) and therefore lead to a possible dysfunction of diaphragmatic breathing. The insertions of this muscle could affect the structure of the thoracic vertebrae but also lumbar and psoas (hyperlordosis, pelvis rotations, etc.) [9,10]. 

The relationship between the hypoglossal nerve and the phrenic nerve should also be considered for the presence of an anastomosis between the two nerves, and because both receive anastomotic filaments from the cervical ganglia. Both have proprioceptive and sensitive motor fibers. This explains secondary hypertonic cervicalgia reflected in patients with gallbladder stones, marked steatosis, or cirrhosis [11]. The cranio-maxillofacial complex has close links with the entire rib cage (visceral chain anteriorly and vertebral posteriorly); all these structures have a common membranous origin and therefore there is a link in the harmonic or pathological development. The hyoid bone has a role in supporting the visceral chain and maintaining the patency of the airways and preventing collapse. The jaw has a fundamental role in breathing. A fracture of the symphysis and the drop-in tone of the genioglossus during narcosis testify to the importance of the jaw in breathing. Many repercussions could manifest themselves on the jaw or on its direction of growth when the respiratory function is altered. These muscles participate in the formation of cervical lordosis, while the paravertebral muscles oppose it. Even the genioglossus plays an important role in ventilation by involving the mandibular condyles; its insertion is the parasymphysis and it undergoes all the variations of the respiratory cycle. Its tone increases during inspiration, and its activity is transmitted mechanically through the fundamental line from the symphysis to the condyles. These therefore grow on the basis of the growth rates for the whole organism, but also undergo changes in the impulses of the genioglossus committed to maintaining the airways. Every time there is a mechanical obstruction at the rhino-pharynx, therefore in the condylar proximity, a diminished growth thrust and a different direction of development is achieved at this level (e.g., in Pierre Robin’s syndrome) [12,13,14,15,16].

There are situations of respiratory difficulties not due to pathologies developed in the first years of life, but due to morphological characteristics that move away from harmonic growth. One of these is the reduction of the upper incisor tract. The incisors play a fundamental role in the development of the maxilla: the formation of the crypts create a tension on the alveolar bone that is opposed to that of the peri-oral musculature. The sketches of the incisors take part in the development of the premaxilla and the space that closes the lower part of the nasal cavities. They also contribute to forming the suture between the premaxilla and maxilla, the incisor-canine suture, and delimit the front and side of the maxillary arch. The balance of internal and external forces that determine the formation of the premaxilla end when the incisors erupt (at 6–7 years of age), and this is the moment when the suture between the premaxilla and maxilla begins to close, which will be completed between 12 and 18 years of age. When the permanent incisors erupt, the premaxilla no longer expands transversely as does the lower part of the nasal orifices [17,18,19,20,21,22,23,24].

The temporal muscle, with its insertion in the coronoid process, also performs another task, and that is to keep the jaw hanging in a rest position with the orientation that the jaw must have (i.e., with the horizontal occlusal plane) [25,26,27,28,29,30]. However the explanation may seem simplistic, and logically it is linked to a more complex mechanism involving other organs, but basically the concept remains this: the temporal muscle is not equipped with its own intelligence and even less the jaw, but both are part of a system that performs multiple functions, such as talking, chewing, swallowing, breathing, and maintaining the rest position with the least expenditure of energy. The increase in tension in one of the elements of the structure will produce an increase in tension in other elements, even those on the opposite side and those that are in other districts. By increasing mechanical stress, there are many elements that should orient themselves in the direction of the applied force; the result is a linear rigidity of the structure. By applying excessive tension, the structure could collapse in several points far from that of the application, for example, in previously affected sites [31].

The conclusion is that the human body is a self-balanced and efficient unit in which information is distributed globally from the microscopic level up to the macroscopic level. The mandibular dysfunctions that could give interference at the postural level are those that result in unilateral chewing or centric chewing. For example, starting from the prevalence of masseter, temporal (anterior beam), internal pterygoid, areas of fascial density are created that progressively affect the ipsilateral fascial and flexion chains of the crusades, with consequent postural adaptations at the cervical and shoulder-humeral girdle level that reflect the reduction in vertical size found at the occlusal level on the prevalent chewing side [32,33,34,35].
